# Double-Criteria Active Learning for Multiclass Brain-Computer Interfaces

**DOI:** 10.1155/2020/3287589

**Published:** 2020-03-10

**Authors:** Qingshan She, Kang Chen, Zhizeng Luo, Thinh Nguyen, Thomas Potter, Yingchun Zhang

**Affiliations:** ^1^Institute of Intelligent Control and Robotics, Hangzhou Dianzi University, Hangzhou, Zhejiang 310018, China; ^2^Department of Biomedical Engineering, University of Houston, Houston, TX 77204, USA

## Abstract

Recent technological advances have enabled researchers to collect large amounts of electroencephalography (EEG) signals in labeled and unlabeled datasets. It is expensive and time consuming to collect labeled EEG data for use in brain-computer interface (BCI) systems, however. In this paper, a novel active learning method is proposed to minimize the amount of labeled, subject-specific EEG data required for effective classifier training, by combining measures of uncertainty and representativeness within an extreme learning machine (ELM). Following this approach, an ELM classifier was first used to select a relatively large batch of unlabeled examples, whose uncertainty was measured through the best-versus-second-best (BvSB) strategy. The diversity of each sample was then measured between the limited labeled training data and previously selected unlabeled samples, and similarity is measured among the previously selected samples. Finally, a tradeoff parameter is introduced to control the balance between informative and representative samples, and these samples are then used to construct a powerful ELM classifier. Extensive experiments were conducted using benchmark and multiclass motor imagery EEG datasets to evaluate the efficacy of the proposed method. Experimental results show that the performance of the new algorithm exceeds or matches those of several state-of-the-art active learning algorithms. It is thereby shown that the proposed method improves classifier performance and reduces the need for training samples in BCI applications.

## 1. Introduction

Brain-computer interfaces (BCIs) are systems that allow users to control external devices via observed brain activity, without relying on peripheral nerve or muscle activity [1]. The most common and useful BCIs are constructed using noninvasive brain activity recording techniques, such as electroencephalography (EEG) [2]. While EEG has become widely used for medical monitoring, rehabilitation, neuroprosthesis, and other healthcare applications [3–5], the data acquisition process can be lengthy and exhaustive for users [6]. In addition, EEG signals often vary over the course of an experiment due to both biological and technical causes, including subject-specific anatomical differences, intersession variability, and the attentional drift of subjects [7]. Consequently, users must often undergo a long data collection process to train a suitable BCI system. This poses a prohibitive burden for individuals with paralysis or a severely injured central nervous system, making it a major hurdle for therapeutic applications. It is therefore of the utmost importance that developed BCI systems achieve efficient and robust performance with as few samples as possible.

One approach that has been effectively applied to cases with limited training sets is the introduction of active learning (AL) to the BCI calibration procedure. AL queries the class labels of informative samples within the unlabeled sample space to maximize the efficiency of the learning model, and its application greatly reduces the complexity of training samples without any obvious loss of classification accuracy [8]. In essence, AL is an iterative sampling and labeling procedure. On each iteration, AL extracts the sample or batch of samples that are most valuable for improving the current classification model from the unlabeled data pool, and these samples are then manually labeled. The greatest challenge for AL methods is identifying the most informative samples so that the maximum prediction accuracy can be achieved. A number of sample-selection criteria have then been applied to this task, including (1) query-by-committee (QBC), in which several distinct classifiers are used and the selected samples are those with the largest difference between the labels predicted by different classifiers [9–11]; (2) margin uncertainty sampling, wherein the samples are selected according to the maximum uncertainty based on their respective distances from the classification boundaries [12, 13]; (3) max-entropy sampling, which uses entropy as the uncertainty measure via probabilistic modeling [14, 15]; and (4) diversity sampling, which prefers selecting representative samples [16].

Over the past few decades, many supervised learning models have been adopted as baseline classifiers for AL, including linear discriminate analysis (LDA) [12, 17], support vector machine (SVM) [18, 19], artificial neural network (ANN) [20], and extreme learning machine (ELM) [21, 22]. Among these, the ELM has shown a high learning speed and good generalizability in preliminary testing. Additionally, it can be directly applied to both two-class and multiclass classification. To date, few studies have attempted to introduce AL algorithms into the ELM framework, although these have shown the method to be competitive with active SVMs [13, 14, 23]. Specifically, Yu et al. [13] proposed an active learning method called AL-ELM with the goal of saving training time, and results showed a classification performance comparable to that of AL-SVM [18]. Zhang and Er [23] then introduced the SEAL-ELM method by combining the online sequential ELM (OS-ELM) with AL, yielding a higher classification accuracy than offline combinations of AL and SVM on most test datasets. Regrettably, these existing active ELMs only consider a single-querying strategy, leaving space for improvement. The intuitive next step was to then introduce multiple querying strategies to select desirable samples. In fact, researchers have tried to combine two strategies in AL with base classifier SVM, with each performing better than their single-query counterparts [24–26]. At present, however, few implementations of active learning with ELM have been explored and applied for motor imagery- (MI-) based BCI systems [8, 13].

The present investigation intends to fill this gap by combining a two-query AL algorithm with an ELM and testing the method in a BCI application. A well-defined, general framework for active learning is thereby developed in a manner that accounts for both informativeness and representativeness in a multiclass situation. First, an uncertainty sampling strategy is adopted to select a relative large number of samples using the base ELM classifier. The degree of diversity between labeled training data and previously selected, unlabeled samples is then assessed, along with the degree of similarity between the unlabeled samples. Finally, highly informative and representative samples are used to update the ELM classifier through the introduction of a tradeoff parameter. The method is then tested on several benchmark datasets, along with a multiclass MI EEG dataset from BCI Competition IV Dataset 2a. Results demonstrate that the performance of the new method compares favorably with that of existing AL approaches.

Compared to existing ELM-based active learning algorithms, the new method has several noteworthy aspects:

1. Considering that the use of a single uncertainty strategy may not take full advantage of the abundant information with unlabeled data, the AL-ELM algorithm is extended to combine two querying strategies (uncertainty and diversity) in order to select the most valuable samples from the unlabeled EEG data pool.
2. The proposed algorithm provides a straightforward and meaningful way to measure representativeness by assaying two kinds of similarity: the similarity between a query sample and the labeled dataset, and the similarity between any two possible query samples. Employing this modified diversity strategy can help isolate highly representative samples during the active learning process.

## 2. Background Knowledge

### 2.1. Active Learning

Active learning methods typically comprise five basic components: **L**, **U**, **T**, **Q**, and **S**. **L** is the limited labeled dataset, **U** is the pool of samples/instances that contains abundant unlabeled instances, **T** is the classification model trained by **L**, **Q** is a query strategy to select the most valuable instances from **U**, and **S** is a human annotator that labels the selected instances correctly. AL is an iterative procedure that gradually adds the most important samples, queried by **Q** and labeled by **S**, from **U** to **L** to update the classification model **T**. The iterative AL process will continue in this manner until a predefined criterion is met. The ability to identify both an excellent classification model **T** and an effective query strategy **Q** is highly important for active learning algorithms.

Depending on the number of querying samples at each iteration, AL can be divided into two groups: stream-based AL and pool-based AL. In stream-based AL, the learner can only access one sample per iteration, while pool-based AL allows the learner to select a batch of samples during each iteration. Adjusting the selection method and number of queried samples then creates different AL algorithms, such as the QBC strategy, the uncertainty strategy, and the diversity strategy.

### 2.2. Basic ELM

Single-hidden-layer feedforward neural networks (SLFNs) are capable of universal approximation [21]. Consider a dataset containing *N* training samples, {*X*, *Y*}={*x*_*j*_, *y*_*j*_}_*j*=1_^*N*^, with the input *x*_*j*_=[*x*_*j*1_, *x*_*j*2_,…,*x*_*jp*_]^*T*^ ∈ *ℝ* ^*p*^ and a corresponding desired output of *y*_*j*_=[*y*_*j*1_, *y*_*j*2_,…,*y*_*jq*_]^*T*^ ∈ *ℝ* ^*q*^, where *p* and *q* represent the respective dimensions and T denotes a transpose operation. Assuming that *M* is the number of hidden neurons, the output function of the SLFNs is mathematically modeled as

(1) $$\begin{array}{c} y_{j}=\sum_{i=1}^{M}\beta_{i}g\left(a_{i}^{\text{T}}x_{j}+b_{i}\right),\;j=1,\ldots ,N, \end{array}$$

where **β**_*i*_=[β_*i*1_, β_*i*2_,…,β_*iq*_]^T^ ∈ **ℝ** ^*q*^ is the weight vector that connects the *i*-th hidden neuron to the output neurons, **a**_*i*_=[*a*_*i*1_, *a*_*i*2_,…,*a*_*ip*_]^T^ ∈ **ℝ** ^*p*^ is a randomly chosen input weight vector connecting the *i*-th hidden neuron to the input neurons, *b*_*i*_ ∈ **ℝ**(*i*=1,…, *M*) is a randomly chosen bias of the *i*-th hidden node, and *g*(•) is the activation function, which can be any nonlinear piecewise continuous function (such as a sigmoid function or Gaussian function).

For convenience, equation (1) can be rewritten in matrix notation as

(2) $$\begin{array}{c} Y=H\beta , \end{array}$$

where **Y**=[**y**_1_, **y**_2_,…,**y**_*N*_]^T^ ∈ **ℝ** ^*N*×*q*^ is the expected network output, **β**=[**β**_1_, **β**_2_,…,**β**_*M*_]^*Τ*^ ∈ **ℝ** ^*M*×*q*^ denotes the weight of output layer, and **H** is the hidden layer output matrix which is defined as

(3) $$\begin{array}{c} H={\left[\begin{array}{ccc} g\left(a_{1}^{\text{T}}x_{1}+b_{1}\right) & \ldots & g\left(a_{M}^{\text{T}}x_{1}+b_{M}\right)\\ \ldots & \ldots & \ldots \\ g\left(a_{1}^{\text{T}}x_{N}+b_{1}\right) & \ldots & g\left(a_{M}^{\text{T}}x_{N}+b_{M}\right) \end{array}\right]}_{N\times M}. \end{array}$$

Unlike SLFNs, which require that the parameters of hidden neurons are adjusted during training, ELM adopts randomly generated hidden layer parameters and a tuning-free training strategy [22]. Even with these random hidden node parameters, ELM maintains the universal approximation capability of SLFNs [21]. The ELM training then aims to find suitable network parameters to minimize the approximation error ‖**H** **β** − **Y**‖_2_. To achieve better generalization performance, a regularization parameter *c* is introduced in [27], with its corresponding objective function given as

(4) $$\begin{array}{c} \underset{\beta }{\min}\frac{1}{2}{\left\|\beta \right\|}_{2}^{2}+\frac{c}{2}{\left\|H\beta -Y\right\|}_{2}^{2}, \end{array}$$

where ‖*·*‖_2_ denotes the *l*_2_-norm of a matrix or a vector. We can obtain the output weight vector **β** using the Moore-Penrose principle. The solution of equation (4) is then **β**=((**I**/*c*)+**H** ^T^ **H**)^−1^ **H** ^T^ **Y** if *N* > *M*, and **β**=**H** ^T^((**I**/*c*)+**H** **H** ^T^)^−1^ **Y** if *N* < *M*.

## 3. The D-AL-ELM Method

In this section, we present a novel active learning algorithm, D-AL-ELM, that incorporates both the uncertainty and diversity strategies into consecutive steps. This identifies the most valuable, informative instances, which can then be selected to update the baseline classifier ELM during each learning round.

### 3.1. Discriminative Information by the Uncertainty Criterion

The uncertainty criterion is used to measure the informativeness of each sample. Uncertain samples which lie along the boundaries of different classes carry more information and play a more significant role in the construction of a classifier. In this implementation, the best-versus-second-best (BvSB) strategy is adopted to estimate the uncertainty of each sample. The BvSB strategy is based on a calculation of posterior probability, which considers the difference in probability values between the two classes with the highest estimated probabilities [28]. The outputs of the ELM then approximate the posterior probabilities of the different classes [13]. To do this, a sigmoid function is used to construct a mapping relationship between the real outputs of the ELM and the posterior probabilities, which is described as

(5) $$\begin{array}{c} p\left(y=1\left|\;\right.f_{i}\left(x\right)\right)=\frac{1}{1+\exp\left(-f_{i}\left(x\right)\right)}, \end{array}$$

where *f*_*i*_(*x*) denotes the actual output of the *i* − th output node corresponding to the time instance *x*. In practice, equation (5) is only applied to two-class problems, such that the sum of the converted posterior probabilities for the instance *x* is always 1. However, application in multiclass problem may create a summed posterior proximity that exceeds 1, so calculated probabilities were normalized using the following formula:

(6) $$\begin{array}{c} \bar{p}\left(y=1\left|\;\right.f_{i}\left(x\right)\right)=\frac{p\left(y=1\left|\;\right.f_{i}\left(x\right)\right)}{\sum_{j=1}^{q}p\left(y=1\left|\;\right.f_{j}\left(x\right)\right)}, \end{array}$$

where *p*(*y*=1|*f*_*i*_(*x*)) is the original probability of the *i* − th class.

Based on the above parameters, the BvSB strategy for each sample *x* can be expressed as

(7) $$\begin{array}{c} f{\left(x\right)}^{BvSB}=p\left(y_{\text{best}}\left|\;\right.x\right)-p\left(y_{\text{second}-\text{best}}\left|\;\right.x\right), \end{array}$$

where *p*(*y*_best_ *|* *x*) and *p*(*y*_second−best_ *|* *x*) are the largest and second largest posterior probabilities of x, respectively. It should be noted that *f*(**x**)^*BvSB*^ values are inversely related to the amount of uncertainty in a sample, with smaller values indicating greater uncertainty.

### 3.2. Representative Information by the Diversity Criterion

The selection of redundant or overly similar samples is of little use when attempting to construct a robust classifier. It is therefore necessary to use a diversity criterion to select a batch of samples which are diverse in nature. A feasible way of measuring the diversity of uncertain samples is the cosine angle distance. Following this approach, the similarity between two samples *x*_*i*_ and *x*_*j*_ is given by

(8) $$\begin{array}{c} S\left(x_{i},x_{j}\right)=\left|\;\right.\cos\left(x_{i},x_{j}\right)\left|\;\right.=\frac{\left|x_{i}\cdot x_{j}\right|}{\left\|x_{i}\right\|\left\|x_{j}\right\|}. \end{array}$$

As can be seen from equation (8), the similarity *S*(*x*_*i*_, *x*_*j*_) between the two samples *x*_*i*_ and *x*_*j*_ is small if these two samples are far from each other, and vice versa.

Suppose a batch of samples **W**={*w*_1_, *w*_2_,…, *w*_*n*_}. If the value of *max*_*i*=1,…,*n*_ *S*(*x*, *w*_*i*_) is small, then the new sample *x* is diverse from the samples in **W**. The similarity between a new sample *x* and **W** is defined as

(9) $$\begin{array}{c} div\left(x,W\right)=\underset{w_{j}\in W}{max}S\left(\text{x},{\text{w}}_{j}\right). \end{array}$$

Note that a smaller *di* *v*(*x*, **W**) value implies more diversity between *x* and **W**.

In order to avoid selecting highly redundant samples, a novel diversity criterion is defined by combining the similarity between a query sample and the labeled set, and the similarity between any two candidate query samples at the same time. This calculation is given by

(10) $$\begin{array}{c} div\left(w_{i}\right)=div\left(w_{i},W\right)+div\left(w_{i},L\right), \end{array}$$

where *div*(*w*_*i*_, **W**) represents the diversity between the sample *w*_*i*_ and the candidate set **W** (apart from *w*_*i*_), and *div*(*w*_*i*_, **L**) represents the diversity between the sample *w*_*i*_ and the labeled training set **L**.

### 3.3. Proposed D-AL-ELM Algorithm

The BvSB sampling method is a highly effective strategy for sample selection in active learning. Unfortunately, the BvSB may also select some uncertain samples which contain highly redundant information, which reduces the information available for classification. To address this problem, optimal samples were selected for classification. An ideal sample would not only furnish significant information for the classifier but also show diversity from the candidate unlabeled set and a minimal amount of redundancy within the labeled set.

The specific steps for each iteration of the D-AL-ELM algorithm are as follows:

Step 1: the BvSB strategy is adopted to select the *h* most uncertain samples from the unlabeled samples pool **U**.
Step 2: let *h* represent the most uncertain samples, denoted by **W** = {*w*_1_, *w*_2_,…, *w*_*h*_} ⊆ **U**, and **S**_*m*_ be an arbitrary subset containing *m*(*m* ≤ *h*) samples selected from **W**. Two evaluations are then performed, including the diversity from the labeled set **L** and the candidate set **S**_*m*_, and the similarity to the samples in **S**_*m*_.
Step 3: combining the discriminative and representative parts, the following formulation is obtained to select the *m* samples which are uncertain and diverse from each other:
  (11) $$\begin{array}{c} {\hat{\text{x}}}^{BvSB-div}=\arg\underset{{\text{s}}_{i}\in S_{m}}{\min}\left\{\lambda f{\left(s_{i}\right)}^{BvSB}+\left(1-\lambda \right)\left(div\left(s_{i},S_{m}\right)+\;div\left(s_{i},L\right)\right)\right\}\text{,} \end{array}$$

where *λ* is a tradeoff parameter that can balance the informativeness and representativeness criteria, and **L** is the labeled training set. ${\hat{x}}^{BvSB-div}$ denotes the unlabeled sample that will be annotated and then included into the labeled training dataset for updating the ELM classifier.

The implementation of the proposed method is summarized in Algorithm 1.

In order to quantitatively evaluate the quality of each learning algorithm, area under the learning curve (ALC) [13] was calculated as a performance metric, which is described as

(12) $$\begin{array}{c} \text{ALC}=\sum_{i=0}^{N_{iter}-1}\frac{y_{i}+y_{i+1}}{2N_{iter}}, \end{array}$$

where *N*_*iter*_ denotes the number of learning iterations and *y*_*i*_ denotes the classification accuracy at the *i*-th learning round, such that ALC ∈ [0,1]. It is noted that the larger the ALC value, the better the performance of the learning algorithm.

## 4. Experimental Results and Discussions

In this section, several experiments were performed on benchmark datasets and multiclass MI EEG datasets to evaluate the performance of the proposed D-AL-ELM method, in comparison with the other state-of-the-art approaches, including passive learning-based ELM, AL-ELM [13], and entropy-based ELM [14]. All methods were implemented using the MATLAB 2014b environment on a computer with a 2.5 GHz processor and 4.0 GB RAM.

### 4.1. Experiments on the Benchmark Datasets

#### 4.1.1. Description of the Benchmark Datasets

A series of experiments were performed to evaluate the D-AL-ELM algorithm on 9 benchmark datasets from the KEEL dataset [29] and UCI dataset repositories [30]. Datasets included both binary and multiclass classification problems. As in [13], each raw dataset was divided into three parts: a small initial labeled set, a large unlabeled set, and a testing set. Testing instances comprised 50% of the total number of samples, while the percentage of initially labeled instances was assigned based on the size of the raw dataset and the number of categories. Detailed information regarding these datasets is presented in Table 1.

#### 4.1.2. The Compared Algorithms, Parameter Settings, and the Performance Metric

In our experiments, we compare the proposed method with other state-of-the-art learning algorithms, including the following:

1. PL-ELM: a passive learning algorithm that randomly selects some instances from the unlabeled set to train the initial classifier
2. AL-ELM: a batch-mode active learning method based on ELM that uses the margin sampling strategy to select most uncertain examples for labeling [13]
3. ELM-Entropy: querying discriminative samples through entropy measures [14]

In this study, the ELM adopted a sigmoid function as the activation function on the hidden level. A grid search based on tenfold cross-validation was then used to find the optimal number of hidden nodes *M* in the initial labeled set. For the regularization parameter *c*, a leave-one-out (LOO) cross-validation strategy was adopted based on the minimum *MSE* ^*PRES*^ to find the optimal parameter value [31]. The optimal parameters *M* and *c* were determined from *M* ∈ {10,20,…, 200} and *c* ∈ {*e* ^−5^, *e* ^−4.9^,…, *e* ^5^} on all the datasets except for the Letter dataset, where the parameter *M* was searched among {100,200,…, 1000}. Additionally, the tradeoff parameter *λ* ∈ {0.1, 0.2,…, 0.9} for equation (10) was chosen by grid search when *M* and *c* were fixed through the aforementioned methods. It should be noted that the ELM parameter selection process was implemented in the same manner for all four methods.

Parameter details are shown in Table 2. It should be noted that the regularization parameter *c* was automatically identified using the LOO cross-validation and was not fixed during the learning process (thus, not shown in Table 2).

The batch mode was adopted to add new labeled instances. For the proposed D-AL-ELM method, *h* samples were first selected from the unlabeled set using equation (7), and then *m* samples were selected from the *h* samples using equation (11) and added to the labeled set for each iteration. In this experiment, *h* was empirically set to *h*=5*m* while *m* was 5% of the total instances in the original unlabeled set for 8 of the 9 datasets (except Letter). For the Letter dataset, *m* was 1% of the total instances in the original unlabeled set and *h* was set to *h*=2*m*. These parameters were chosen to decrease the labeling cost, considering the size of the raw dataset and the number of categories.

To provide a fair comparison, all four methods queried *m* instances on each iteration. For each dataset, the procedure was stopped when the prediction accuracy stabilized or the number of selected samples was greater than 80% of the original unlabeled set. Additionally, to ensure the validity of experimental results, ten runs were performed for each learning method in each experiment, and average results were calculated.

#### 4.1.3. Comparisons with Relevant State-of-the-Art Algorithms

**Figure 1:**
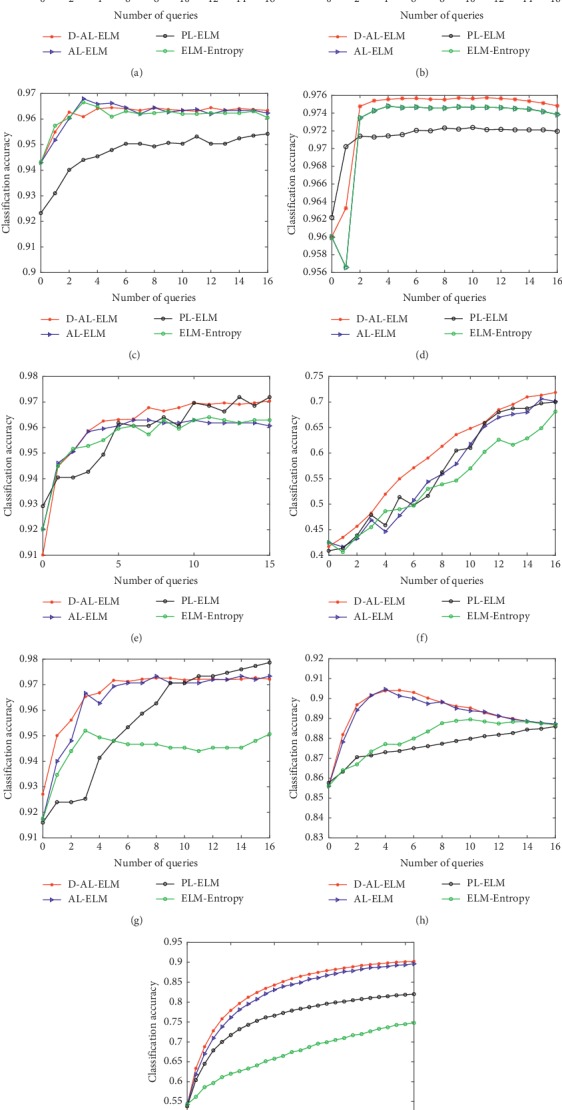
The learning curves of the four different learning algorithms on 9 benchmark datasets. (a) Liver. (b) Diabetes. (c) Wdbc. (d) Twonorm. (e) Wine. (f) Hayes-Roth. (g) Iris. (h) Segment. (i) Letter.

Figure 1shows the trends of classification accuracy for the classifiers when trained by increasing numbers of data points across the various datasets. The results show that the proposed D-AL-ELM algorithm yielded the highest accuracy of all four methods on most of datasets (excepting the Wine and Iris datasets) at the last learning round. Specifically, the proposed method performed better than the remaining three methods over the majority of the active learning period for the Twonorm, Hayes-Roth, and Letter datasets. Moreover, the D-AL-ELM yielded the fastest learning rate over the first few iterations of the learning process for most datasets. This phenomenon indicates that the new method begins by effectively identifying the most informative and representative samples, unlike the other algorithms. Additionally, the ELM-Entropy approach generally yielded lower accuracy in multiclass classification, failing to surpass the PL-ELM on the Wine, Hayes-Roth, Iris, and Letter datasets. Another interesting observation was that the performance tended to degrade at a certain interval on the Segment dataset. It was considered that the Segment dataset may have a more irregular data structure, confounding the BvSB strategy and deteriorating the result. In cases such as this, a more adaptive stop criterion should be designed to stop the learning program at a more appropriate right time, before output degrades.

Table 3 presents the mean classification accuracies of the four methods across the 9 datasets during the learning process. The ALC values for the four methods are further compared in Table 4. The results shown in Tables 3 and 4 indicate that the D-AL-ELM method yielded the best performance among all datasets for the tested methods. As in [13], the ALC metric not only was related to the learning velocity but also had close relationship to the quality of the learning model. The proposed D-AL-ELM outperformed the other methods in terms of ALC, with the AL-ELM performing second best, with an accuracy close to that of the D-AL-ELM on the Wdbc and Segment datasets. For the Wdbc dataset, although the proposed method had a slightly higher ALC value than the AL-ELM, both algorithms yielded the same mean accuracy for the overall learning process.

Finally, Table 5 reports the average time for the learning stage of each algorithm across all datasets. As expected, the PL-ELM was the fastest method because it lacked any criteria for the evaluation of samples. The proposed D-AL-ELM required slightly more learning time than AL-ELM and ELM-Entropy, since it computed both informativeness and representativeness of each instance. Considering the improvement of classification performance, this extra time may be deemed an acceptable tradeoff.

#### 4.1.4. Analysis of Effect of Different Batch Size Values

In this experiment, the performance of the proposed active learning method was further evaluated using different batch sizes (i.e., *h* and *m* values).

**Figure 2:**
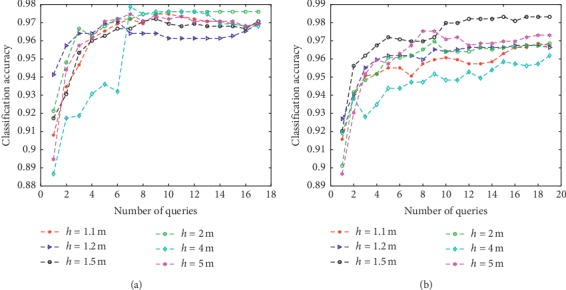
The learning curves of the proposed algorithm with different h values on Iris and Wine. (a) Iris. (b) Wine.

**Figure 3:**
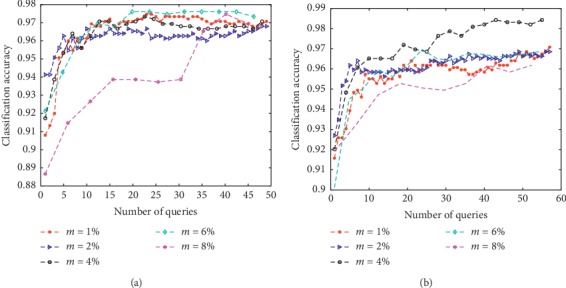
The learning curves of the proposed algorithm with different m values on Iris and Wine. (a) Iris. (b) Wine.

The new method was tested with different querying sizes by varying the values of *h* and *m*, respectively. The remaining experimental settings were the same as in earlier experiments and testing was conducted on two benchmark datasets: Iris and Wine. The *M* and *λ* parameters were set as *M*=100,  *λ*=0.5 to observe the performance with different batch sizes. Results are reported in Figures 2 and 3. In Figure 2, *m* was fixed at 5% of the total number of instances in the original unlabeled set and *h* was chosen from a candidate set {*h*=1.1*m*, 1.2*m*, 1.5*m*, 2*m*, 4*m*, 5*m*}. In Figure 3, *h* was fixed at the value of 2*m* and *m* was chosen from {1%, 2%, 4%, 6%, 8%}. It can be seen from Figure 2 that learning rates at the start of the curve increased with higher *h* values. Performance on Iris was less sensitive to the *h* value when enough instances were queried, and learning curves tended to be similar when query numbers and *h* values were large. In contrast, performance on the Wine dataset was more sensitive to *h*. This may be a result of the Wine dataset having a more complex distribution, which is difficult to capture. Although the D-AL-ELM performed differently on the two datasets, relatively larger *h* values were consistently able to obtain favorable performance. On the other hand, this increase in *h* value leads to a greater computational burden. Figure 3 shows the effects of different values of *m* on the Iris and Wine datasets. From this, it was observed that convergence can be more easily achieved with small *m* values. Alternatively, when *m* is large, more instances can be learned at each iteration and the number of total iterations greatly reduced, although this boost in performance does not provide substantially increased accuracy. In conclusion, optimizing the *h* and *m* values is not crucial for the D-AL-ELM, as most values yield similar results. It should be noted, however, that larger *h* and *m* are generally recommended.

### 4.2. Experiment on Multiclass MI EEG Data

#### 4.2.1. Description of EEG Datasets

This section further evaluates the performance of the proposed D-AL-ELM method on multiclass MI EEG data from the BCI Competition IV Dataset 2a [32]. This dataset consists of the EEG signals from 9 subjects who performed 4 tasks, including left hand, right hand, foot, and tongue MI. EEG signals were recorded using 22 electrodes. Each subject underwent a training and testing session, each consisting of 288 trials (a total of 576 trials across the two sessions).

#### 4.2.2. Experimental Setup and Parameter Settings

Data preprocessing was first performed on the raw EEG data. For each trial, features were extracted from the time segment lasting from 0.5 s to 2.5 s after the cue instructing the subject to perform MI. Each trial was first band-pass filtered from 8–30 Hz using a fifth-order Butterworth filter. Next, the dimension of the EEG signal was reduced to a 24-dimension feature set using the one-versus-rest common spatial pattern (OVR-CSP) algorithm [33], which is an effective and popular feature extraction method for EEG multiclassification that computes the features that discriminate each class from the remaining classes. Finally, the features extracted by OVR-CSP were discriminated using the different classification methods.

Optimal selection of the *M*, *λ*, and *c* parameters was performed in the same manner described in Section 4.1.2. The number of hidden nodes *M* was searched within {10,20, ..., 150}. For each subject, the first 400 trials were considered as the training set, while the remaining 176 trials were used as the independent testing set [11]. The values for *m* and *h* were set at *m*=10 and *h*=5*m*. Finally, experiments included ten runs for each learning method from which average results were calculated.

#### 4.2.3. Comparisons with Related Algorithms

**Figure 4:**
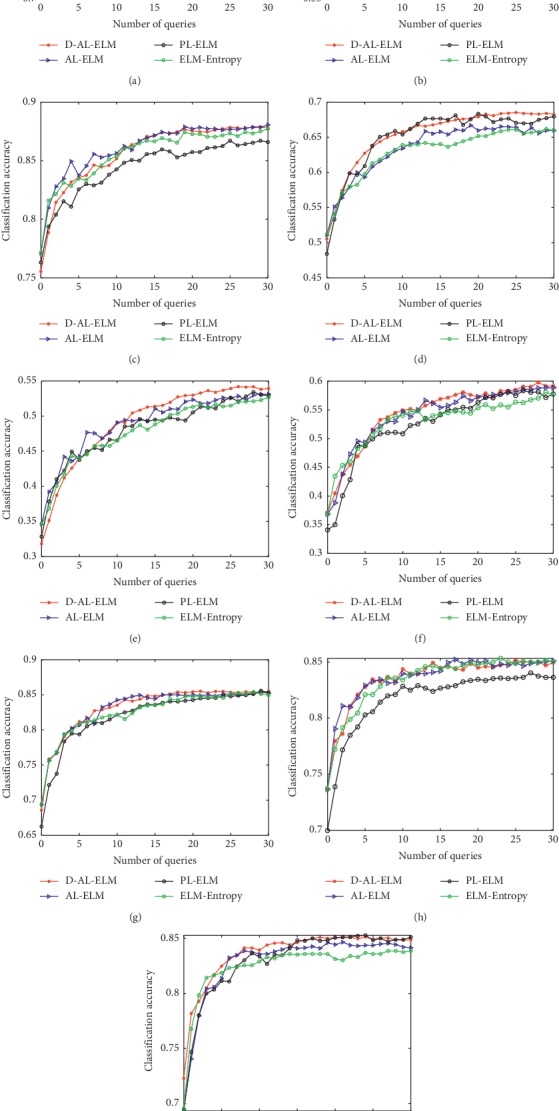
Learning curves of the four different learning algorithms on BCI Competition IV Dataset 2a. (a) S1. (b) S2. (c) S3. (d) S4. (e) S5. (f) S6. (g) S7. (h) S8. (i) S9.

Figure 4 illustrates the trend lines of classification accuracy when methods were applied to different testing datasets, while Table 6 lists the mean classification accuracies of the four methods during the learning process.  Table 7 then provides the ALC results, while Table 8 shows the average running time (s) for the learning stage.

The results show that the performance of D-AL-ELM method is comparable to that of the AL-ELM and better than that of the ELM-Entropy and PL-ELM algorithms for all subjects (except for subject 2 in PL-ELM). Specifically, the proposed method surpassed the AL-ELM approach in 6 of the 9 subjects (1, 2, 4, 5, 6, 9) in terms of the ALC metric. For all 9 subjects, the D-AL-ELM method yielded a mean accuracy of 71.36%, higher than that of AL-ELM (70.92%), ELM-Entropy (70.34%), and PL-ELM (70.51%). These results demonstrate the effectiveness of the D-AL-ELM in selecting both informative and representative instances from unlabeled EEG samples. Additionally, they reveal that the proposed method can calibrate an effective classifier for MI EEG signals without the need for a large number of labeled training samples.

For comparative purposes, Table 8 also provides the average running time of each learning algorithm. Although the D-AL-ELM exhibited slightly longer training time than the other three methods, this may be considered a worthwhile tradeoff for the improved classification performance of the D-AL-ELM.

### 4.3. Discussion

In these experiments, the proposed D-AL-ELM method exhibited excellent performance in both classification accuracy and computational efficiency, as demonstrated on several benchmark datasets and an experimental MI EEG dataset. When compared to a passive learning-based ELM, D-AL-ELM achieved improved performance by effectively extracting the most valuable unlabeled samples. The D-AL-ELM also outperformed the AL-ELM and ELM-Entropy algorithms, which both employed a single-query strategy. Improvement was seen on all nine datasets in Section 4.1, evidencing the ability of the D-AL-ELM to boost overall learning performance by combining the uncertainty and diversity strategies when updating the classifier with the selected samples. In terms of computational efficiency, the slight increase in training time for the D-AL-ELM, as compared to the PL-ELM, AL-ELM, and ELM-Entropy, was negligible in practice, especially when considering the improved classification accuracy. The experimental results then demonstrate that the proposed algorithm can effectively and comprehensively measure the representativeness of samples. Simultaneously, the proposed approach also measures how informative individual examples are, contributing to the improved classifier performance. Combining these factors, suitable instances can be selected for classifier construction.

Finally, the effectiveness of the D-AL-ELM was shown in its application to an experimental multiclass MI task from the BCI Competition IV Dataset 2a. Due to the low signal-to-noise ratio of EEG data, the applied algorithms struggled to generate adequate results. Consequently, hand-designed features were first extracted from the raw EEG data using the OVR-CSP approach, and the different AL algorithms were then used to further extract the unlabeled samples and calibrate a robust classifier. For subjects S1, S3, S7, S8, and S9, the D-AL-ELM yielded an acceptably high mean classification accuracy of over 80% for the whole learning process. Unfortunately, all tested methods performed poorly on subject S5. The proposed algorithm was only able to achieve 49.06% accuracy which, though insufficient, still ranked the highest among the applied methods.

## 5. Conclusion

In this paper, a novel active learning method with ELM, the D-AL-ELM, was developed for multiclassification. This new algorithm combines the uncertainty and diversity strategies and effectively reduces the expense and time cost of obtaining labeled data manually. For each sample, the proposed algorithm employs a BvSB strategy to measure informativeness and the cosine angle distance to measure diversity. The modified diversity measure not only estimates the diversity between the limited labeled training data and previously selected unlabeled samples, but also calculates the similarity among previously selected samples. Experimental results from several benchmark datasets and the multiclass MI EEG data from BCI Competition IV Dataset 2a were then used to verify the efficacy of the proposed D-AL-ELM algorithm. These results indicate that the performance of the proposed algorithm is consistently better than, or at least comparable to, that of other popular active learning techniques. Future work will then aim to develop online learning for the D-AL-ELM [23, 34]. In addition, an adaptive stopping criterion may be applied to promote the efficiency of the D-AL-ELM and improve its abilities for the classification and evaluation of MI EEG signals.

## Figures and Tables

**Algorithm 1:**
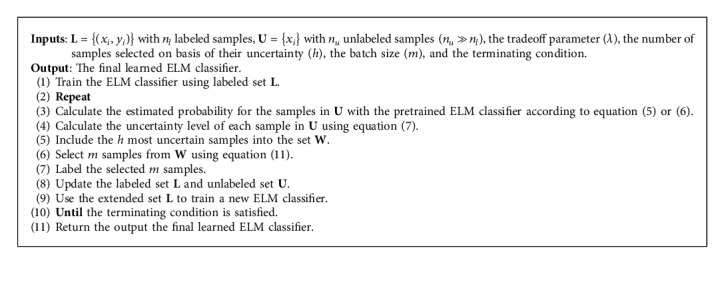
The double-criteria active learning with the ELM algorithm.

**Table 1:** Details of the datasets including the numbers of the corresponding features and samples.

Dataset	Number of			Percentage of initial labeled instances (%)	Percentage of initial unlabeled instances (%)	Percentage of test instances (%)
	Features	Instances	Classes			
Liver	7	345	2	10	40	50
Diabetes	8	768	2	10	40	50
Wdbc	30	569	2	10	40	50
Twonorm	20	7400	2	1	49	50
Hayes-Roth	4	160	3	10	40	50
Iris	4	150	3	10	40	50
Wine	13	178	3	10	40	50
Segment	19	2310	7	10	40	50
Letter	16	20000	26	1	49	50

**Table 2:** Details of the optimal parameter settings for the different datasets using four methods.

Dataset	D-AL-ELM		AL-ELM	ELM-Entropy	PL-ELM
	*M*	*λ*	*M*	*M*	*M*
Liver	110	0.1	110	110	110
Diabetes	110	0.3	110	110	110
Wdbc	200	0.1	200	200	200
Twonorm	120	0.1	120	120	120
Hayes-Roth	100	0.3	100	100	100
Iris	170	0.9	170	170	170
Wine	60	0.7	60	60	60
Segment	200	0.6	200	200	200
Letter	700	0.4	700	700	700

**Table 3:** Mean accuracy results of the learning processes on 9 datasets (%).

Dataset	D-AL-ELM	AL-ELM	ELM-Entropy	PL-ELM
Liver	**67.59**	67.46	67.14	66.14
Diabetes	**76.31**	76.13	76.12	74.60
Wdbc	**96.18**	**96.18**	96.11	94.69
Twonorm	**97.38**	97.25	97.25	97.13
Wine	**96.08**	95.72	95.64	95.79
Hayes-Roth	**59.43**	56.23	54.03	56.56
Iris	**96.65**	96.43	94.44	95.58
Segment	**89.26**	89.17	88.06	87.63
Letter	**82.89**	81.67	67.09	75.76

**Table 4:** ALC comparisons of four methods on 9 datasets.

Dataset	D-AL-ELM	AL-ELM	ELM-Entropy	PL-ELM
Liver	**0.7624**	0.7610	0.7572	0.7447
Diabetes	**0.7640**	0.7624	0.7622	0.7469
Wdbc	**0.9624**	0.9623	0.9616	0.9474
Twonorm	**0.9742**	0.9729	0.9729	0.9715
Wine	**0.9622**	0.9584	0.9573	0.9584
Hayes-Roth	**0.5959**	0.5622	0.5395	0.5663
Iris	**0.9676**	0.9655	0.9450	0.9563
Segment	**0.8939**	0.8929	0.8812	0.8766
Letter	**0.8330**	0.8204	0.6718	0.7606

**Table 5:** Average running time (s) for each learning algorithm.

Dataset	D-AL-ELM	AL-ELM	ELM-Entropy	PL-ELM
Liver	0.9141	0.7531	0.7719	0.7453
Diabetes	1.2031	1.0438	1.0060	0.9719
Wdbc	1.3484	1.2500	1.2828	1.2234
Twonorm	7.9813	5.3047	5.5875	4.8844
Wine	0.5391	0.4625	0.4625	0.4391
Hayes-Roth	0.5109	0.4516	0.4594	0.4203
Iris	0.5250	0.4469	0.4856	0.4313
Segment	3.6578	3.3906	3312	3.3250
Letter	121.8047	115.5203	123.0641	111.4641

**Table 6:** Mean accuracy (%) of the learning process on BCI Competition IV Dataset 2a.

Datasets	D-AL-ELM	AL-ELM	ELM-Entropy	PL-ELM
S1	**83.78**	83.64	83.32	83.76
S2	53.91	52.50	52.33	**54.55**
S3	85.66	**86.06**	85.57	84.39
S4	**65.32**	63.48	62.94	64.95
S5	**49.06**	48.90	47.79	48.00
S6	**54.31**	53.96	52.97	52.36
S7	83.15	**83.34**	82.45	81.96
S8	83.46	**83.56**	83.34	81.71
S9	**83.57**	82.80	82.39	82.91
Mean	**71.36**	70.92	70.34	70.51

**Table 7:** ALC values of the four methods on BCI Competition IV Dataset 2a.

Datasets	D-AL-ELM	AL-ELM	ELM-Entropy	PL-ELM
S1	**81.22**	0.8109	0.8076	81.21
S2	0.5238	0.5100	0.5085	**0.5296**
S3	0.8302	**0.8340**	0.8291	0.8177
S4	**0.6340**	0.6159	0.6105	0.6308
S5	**0.4764**	0.4749	0.4638	0.4662
S6	**0.5276**	0.5241	0.5144	0.5088
S7	0.8066	**0.8085**	0.7996	0.7952
S8	0.8090	**0.8100**	0.8079	0.7923
S9	**0.8103**	0.8032	0.7992	0.8042

**Table 8:** Average running time (s) of each learning algorithm.

Datasets	D-AL-ELM	AL-ELM	ELM-Entropy	PL-ELM
S1	1.7266	1.4625	1.4594	1.4172
S2	2.5125	2.2813	2.3859	2.2469
S3	1.7219	1.4859	1.4578	1.4234
S4	2.0719	1.7891	1.8328	1.8125
S5	2.0781	1.8281	1.8141	1.7719
S6	1.7609	1.4594	1.4094	1.3609
S7	2.4188	2.1969	2.1734	2.1641
S8	1.5562	1.3375	1.3609	1.3078
S9	1.2906	0.9484	0.9563	0.8906
Mean	1.9042	1.6432	1.6500	1.5995

## Data Availability

The BCI Competition IV Dataset 2a was used in our study, which is publicly available via the following link: http://www.bbci.de/competition/iv/.
